# Development of a Novel Nomogram for Predicting Premature Rupture of Membrane in Pregnant Women With Vulvovaginal Candidiasis

**DOI:** 10.3389/fmed.2021.717978

**Published:** 2021-11-15

**Authors:** Lilin Yang, Haikuan Wang, Yanfang Li, Cheng Zeng, Xi Lin, Jie Gao, Songping Luo

**Affiliations:** ^1^Department of Gynecology and Obstetrics, The First Affiliated Hospital, Guangzhou University of Chinese Medicine, Guangzhou, China; ^2^Acupuncture, Moxibustion and Rehabilitation School of Clinical Medicine, Guangzhou University of Chinese Medicine, Guangzhou, China; ^3^Second School of Clinical Medicine, Guangzhou University of Chinese Medicine, Guangzhou, China; ^4^First School of Clinical Medicine, Guangzhou University of Chinese Medicine, Guangzhou, China

**Keywords:** premature rupture of membrane, nomogram, predictor, vulvovaginal candidiasis, pregnant women

## Abstract

**Objective:** The aim of this study was to develop a nomogram to predict the risk of premature rupture of membrane (PROM) in pregnant women with vulvovaginal candidiasis (VVC).

**Patients and methods:** We developed a prediction model based on a training dataset of 417 gravidas with VVC, the data were collected from January 2013 to December 2020. The least absolute shrinkage and selection operator regression model was used to optimize feature selection for the model. Multivariable logistic regression analysis was applied to build a prediction model incorporating the feature selected in the least absolute shrinkage and selection operator regression model. Discrimination, calibration, and clinical usefulness of the prediction model were assessed using the C-index, calibration plot, and decision curve analysis. Internal validation was assessed using bootstrapping validation.

**Results:** Predictors contained in the prediction nomogram included age, regular perinatal visits, history of VVC before pregnancy, symptoms with VVC, cured of VVC during pregnancy, and bacterial vaginitis. The model displayed discrimination with a C-index of 0.684 (95% confidence interval: 0.631–0.737). Decision curve analysis showed that the PROM nomogram was clinically useful when intervention was decided at a PROM possibility threshold of 13%.

**Conclusion:** This novel PROM nomogram incorporating age, regular perinatal visits, history of VVC before pregnancy, symptoms with VVC, cured of VVC during pregnancy, and bacterial vaginitis could be conveniently used to facilitate PROM risk prediction in gravidas.

## Introduction

Premature rupture of membrane (PROM) is defined as a spontaneous rupture of embryonic membrane occurring before the onset of labor. PROM occurs in ~8% of all deliveries (1) and can lead to adverse fetal and neonatal outcomes. Adverse outcomes include: for mothers, intra-amniotic infection, postpartum infection, endometritis; and for newborns, cord prolapse, respiratory distress syndrome, sepsis, intraventricular hemorrhage, and even death.

Vulvovaginal candidiasis (VVC) is a common vaginitis that affects 75% of women at least once in their lifetime (2, 3). More than one fifth of women have experienced recurrent VVC (at least 3 symptomatic episodes in a previous year) (4). In high-income countries, recurrent VVC affects about 138 million women annually, and the economic burden from lost productivity is estimated to be up to billion annually (5). Pregnancy increases the incidence of VVC owing to changes of the internal immune environment of the genital tract.

PROM results from the interaction of many factors (6, 7). However, several studies (8–11) have revealed that genital tract infection is the main cause. As the most common cause of vaginitis, VVC plays a significant role in the incidence of PROM. Although, there have been studies regarding the relationship between VVC and PROM, to date, there is no established method for predicting the probability of PROM in pregnant women with VVC. The purpose of this study was to develop a valid but simple prediction tool to assess the risk of PROM in VVC individuals.

## Patients and Methods

### Patients Study Population

We undertook a study of women with VVC who were hospital in-patients, from January 2013 to December 2020. This study was approved by the Ethical Committee of the First Affiliated Hospital of Guangzhou University of Chinese Medicine. Clinical data were evaluated, and the inclusion criteria were as follows: diagnosed as VVC in gestation no matter whether cured or not, singleton pregnancies, and delivery of pregnancy at 28–42 weeks via vaginal delivery or cesarean. The exclusion criteria were: cases with twins or multiple pregnancies, and termination of pregnancy at <28 weeks or >42 weeks. Patients who had severe cognitive disorders, or had serious physical constraints were excluded. The clinical data came from the Hospital information management system in the First Affiliated Hospital of Guangzhou University of Chinese Medicine. and were categorized as: basic patient demographics (age, education level, occupation, marital status, BMI before pregnancy, and regular perinatal visits); history of gestation (gravidity, parity, number of previous vaginal deliveries, number of previous cesarean sections, and number of previous abortions); past medical history (history of VVC before pregnancy, history of cervix operation); other information in the current pregnancy (gestational week of VVC diagnosis, symptoms with VVC, cured of VVC during pregnancy, antifungal therapy during pregnancy, bacterial vaginitis, mycoplasma, chlamydia, polyhydramnios, macrosomia, and malpresentation). PROM manifests as amniorrhexis prior to the onset of labor. The diagnosis of VVC and VVC cure were both confirmed by laboratory testing. Based on the Guideline of preconception care and prenatal care of China (2018) (12), the minimum number of prenatal times was seven. Missing minimum one time inspection was considered out of regular perinatal visit.

### Statistical Analysis

Statistical analyses were performed using SPSS (Version 22.0) and R software (Version 4.0.3). In R software, Packages (“rms”) and Packages (“rmda”) were operated. All tests were two-sided, and a *P*-value < 0.05 was considered statistically significant. Categorical variables were presented as the number of cases, percentages (%). The least absolute shrinkage and selection operator (LASSO) method, which is suitable for the reduction of high-dimensional data (13, 14) was used to select the optimal predictive features associated with risk factors in pregnant women with VVC. Feathers with non-zero coefficients in the LASSO regression model were selected. Subsequently, multivariable logistic regression analysis was used to build a prediction model by incorporating the features selected in the LASSO regression model. Sociodemographic variables with a *P*-value of ≤ 0.05 were included in the model, whereas variables associated with disease and treatment characteristics were all included (15). All potential predictors were used to develop a prediction model for PROM by using the whole cohort. Afterwards, calibration curves were plotted to assess the calibration of the PROM nomogram. A significant test statistic implied that the model did not calibrate perfectly. To quantify the discrimination performance of PROM, Harrell's C-index was measured. The PROM nomogram was subjected to bootstrapping validation (1,000 bootstrap resamples) to calculate a relative corrected C-index (16). Decision curve analysis was conducted to determine the clinical usefulness of the PROM nomogram by quantifying the net benefit; this was calculated by subtracting the proportion of the patients who were false positive and by weighing the relative harm of forgoing interventions compared with the negative consequence of an unnecessary intervention.

## Results

### Patient Characteristics

A total of 417 patients were enrolled. All the patients were divided into PROM and no PROM groups. Patient data included patient demographics, history of gestation, past medical history, and other information (see Table 1); the incidence of PROM was 33.81%.

**Table 1:** Difference between demographic and clinical characteristics of PROM and no PROM groups.

**Demographic characteristics**	***N*** **%**		
	**PROM (*n* = 141)**	**No PROM (*n* = 276)**	**Total (*n* = 417)**
**Age (years)**			
<35	119 (84.40)	257 (93.12)	376 (90.17)
≥35	22 (15.60)	19 (6.88)	41 (9.83)
**Education level**			
Primary	52 (36.88)	96 (34.78)	148 (35.49)
Higher	89 (63.12)	180 (65.22)	269 (64.51)
**Employment**			
Employed	118 (83.69)	234 (84.78)	352 (84.41)
Unemployed	23 (16.31)	42 (15.22)	65 (15.59)
**Marital status**			
Married	136 (96.45)	270 (97.83)	406 (97.36)
Other marital status	5 (3.55)	6 (2.17)	11 (2.64)
**Regular perinatal visits**			
Yes	128 (90.78)	269 (97.46)	397 (95.20)
No	13 (9.22)	7 (2.54)	20 (4.80)
**BMI before pregnancy**			
<25kg/m^2^	138 (97.87)	271 (98.19)	409 (98.08)
≥25kg/m^2^	3 (2.13)	5 (1.81)	8 (1.92)
**Gravity**			
1	61 (43.26)	119 (43.12)	180 (43.17)
2	52 (36.88)	95 (34.42)	147 (35.25)
3	11 (7.80)	40 (14.49)	51 (12.23)
4	13 (9.22)	12 (4.35)	25 (6.00)
5	3 (2.13)	9 (3.26)	12 (2.88)
6	1 (0.71)	1 (0.36)	2 (0.48)
**Parity**			
0	80 (56.74)	154 (55.80)	234 (55.12)
1	50 (35.46)	95 (34.42)	145 (34.77)
≥2	11 (7.80)	27 (9.78)	38 (9.11)
**Number of previous vaginal delivery**			
0	89 (63.12)	165 (59.78)	254 (60.91)
≥1	52 (36.88)	111 (40.22)	163 (39.09)
**Number of previous cesarean**			
0	133 (94.33)	264 (96.65)	397 (95.20)
≥1	8 (5.67)	12 (4.35)	20 (4.80)
**Number of previous abortion**			
0	106 (72.11)	209 (75.72)	315 (75.54)
≥1	35 (24.82)	67 (24.28)	102 (24.46)
**History of VVC before pregnancy**			
Yes	22 (15.60)	15 (5.43)	37 (8.87)
No	119 (84.40)	261 (94.57)	380 (91.13)
**Gestational week of diagnosis VVC**			
<28w	72 (51.06)	142 (51.45)	214 (51.32)
≥28w	69 (48.94)	134 (48.55)	203 (48.68)
**Symptom with VVC**			
Yes	67 (47.52)	104 (37.68)	171 (41.01)
No	74 (52.48)	172 (62.32)	246 (58.99)
**Antifungal therapy during pregnancy**			
No	6 (4.26)	15 (5.43)	21 (5.04)
Yes	135 (95.74)	261 (94.57)	396 (94.96)
**Cured of VVC during pregnancy**			
No	103 (73.05)	144 (52.17)	247 (59.23)
Yes	38 (26.95)	132 (47.83)	170 (40.77)
**Bacterial vaginitis**			
No	116 (82.27)	245 (88.77)	361 (86.57)
Yes	25 (17.73)	31 (11.23)	56 (13.43)
**Mycoplasma**			
Yes	2 (1.42)	2 (0.72)	4 (0.96)
No	139 (98.58)	274 (99.28)	413 (99.04)
**Chlamydia**			
Yes	2 (1.42)	4 (1.45)	6 (1.44)
No	139 (98.58)	272 (98.55)	411 (98.56)
**Cervix operation**			
Yes	3 (2.13)	3 (1.09)	6 (1.44)
No	138 (97.87)	273 (98.81)	411 (98.56)
**Polyhydramnios**			
Yes	0 (0.00)	0 (0.00)	0 (0.00)
No	141 (100.00)	276 (100.00)	417 (100.00)
**Macrosomia**			
Yes	0 (0.00)	0 (0.00)	0 (0.00)
No	141 (100.00)	276 (100.00)	417 (100.00)
**Malpresentation**			
Yes	8 (5.67)	12 (4.35)	20 (4.80)
No	133 (94.33)	264 (95.65)	397 (95.20)

### Feature Selection

**Figure 1:**
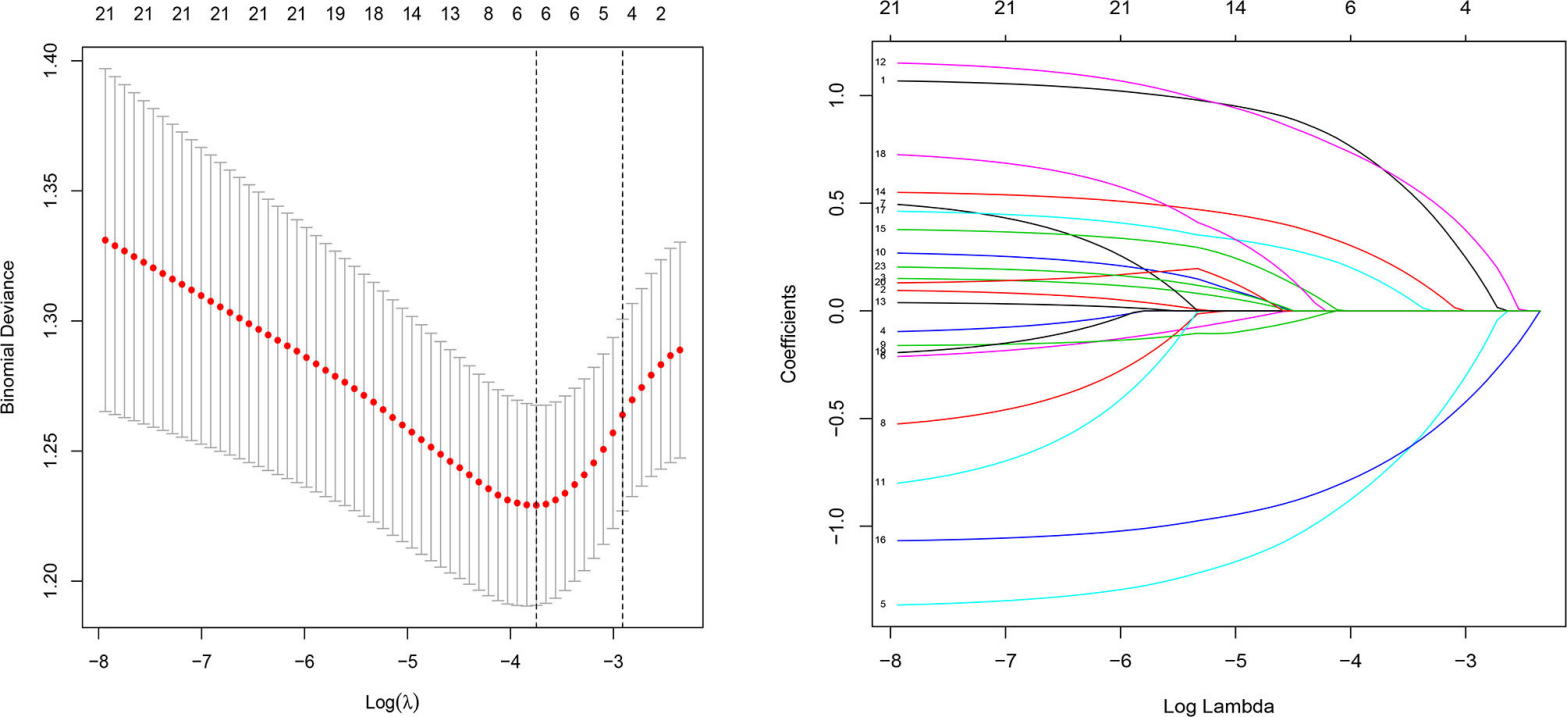
Demographic and clinical feature selection using the LASSO logistic regression model. LASSO, least absolute shrinkage and selection operator; SE, standard error.

Of basic demographic, medical history, and treatment features, 23 features were reduced to 6 potential predictors on the basis of 417 patients in the cohort (Figure 1) and were associated with non-zero coefficients in the LASSO regression model. These features included age, regular perinatal visits, history of VVC before pregnancy, symptoms with VVC, cured of VVC during pregnancy, and bacterial vaginitis (Table 2).

**Table 2:** Prediction factors for PROM in pregnant patients with VVC.

**Intercept ant variable**	**Prediction model**		
	**β**	**Odds ratio (95% CI)**	***P*-value**
Intercept	0.502	1.652(0.566–5.061)	0.362
Age	1.141	3.131(1.521–6.534)	0.002
Regular perinatal visits	−1.358	0.257(0.085–0.740)	0.013
History of VVC before pregnancy	1.027	2.792 (1.322–5.992)	0.007
Symptom with VVC	0.539	1.714 (1.103–2.674)	0.017
Cured of VVC during pregnancy	−1.052	0.349 (0.216–0.554)	<0.001
Bacterial vaginitis	0.449	1.567 (0.832–2.918)	0.159

*β is the regression coefficient. CI, confidence interval*.

### Development of an Individualized Prediction Model

**Figure 2:**
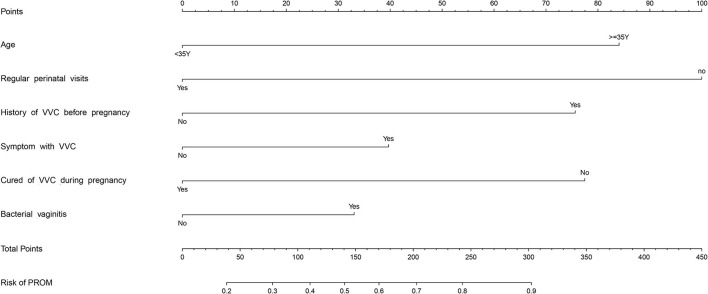
PROM risk assessment tool. Points refers to point for the individual risk factor and add together to the total points.

The results of logistic regression analysis among the use of age, regular perinatal visits, history of VVC before pregnancy, symptoms with VVC, cured of VVC during pregnancy, and bacterial vaginitis are given in Table 2. The model that incorporated the above independent predictors was developed and presented as the nomogram (Figure 2).

### Apparent Performance of the PROM Risk Nomogram in the Cohort

**Figure 3:**
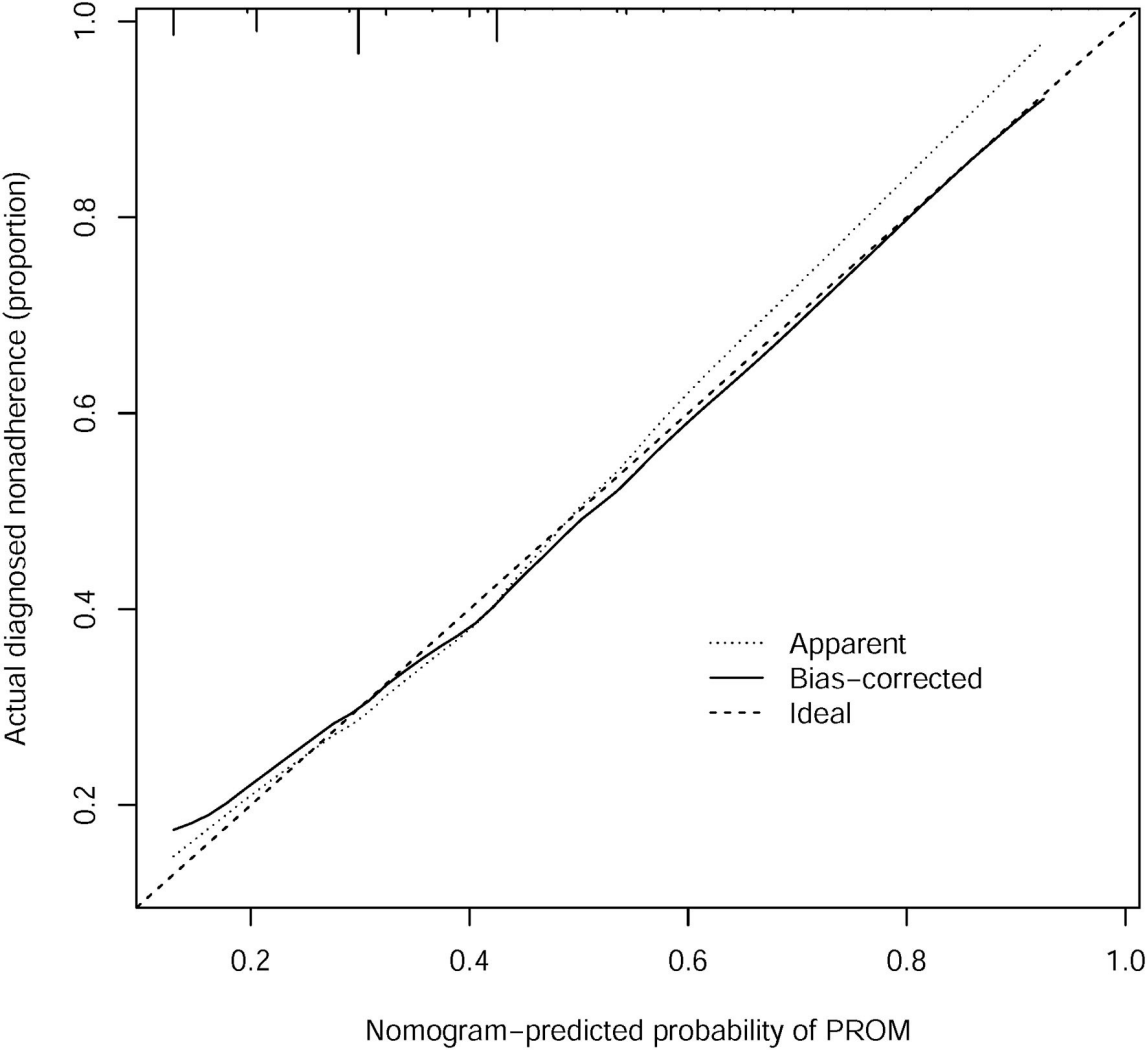
Calibration curves of the PROM nomogram prediction in the cohort.

The calibration curve of the PROM risk nomogram for the prediction of PROM in pregnant women indicated good agreement in this cohort (Figure 3). The C-index for the prediction nomogram was 0.684 (95% confidence interval: 0.631–0.737) for the cohort.

### Clinical use

**Figure 4:**
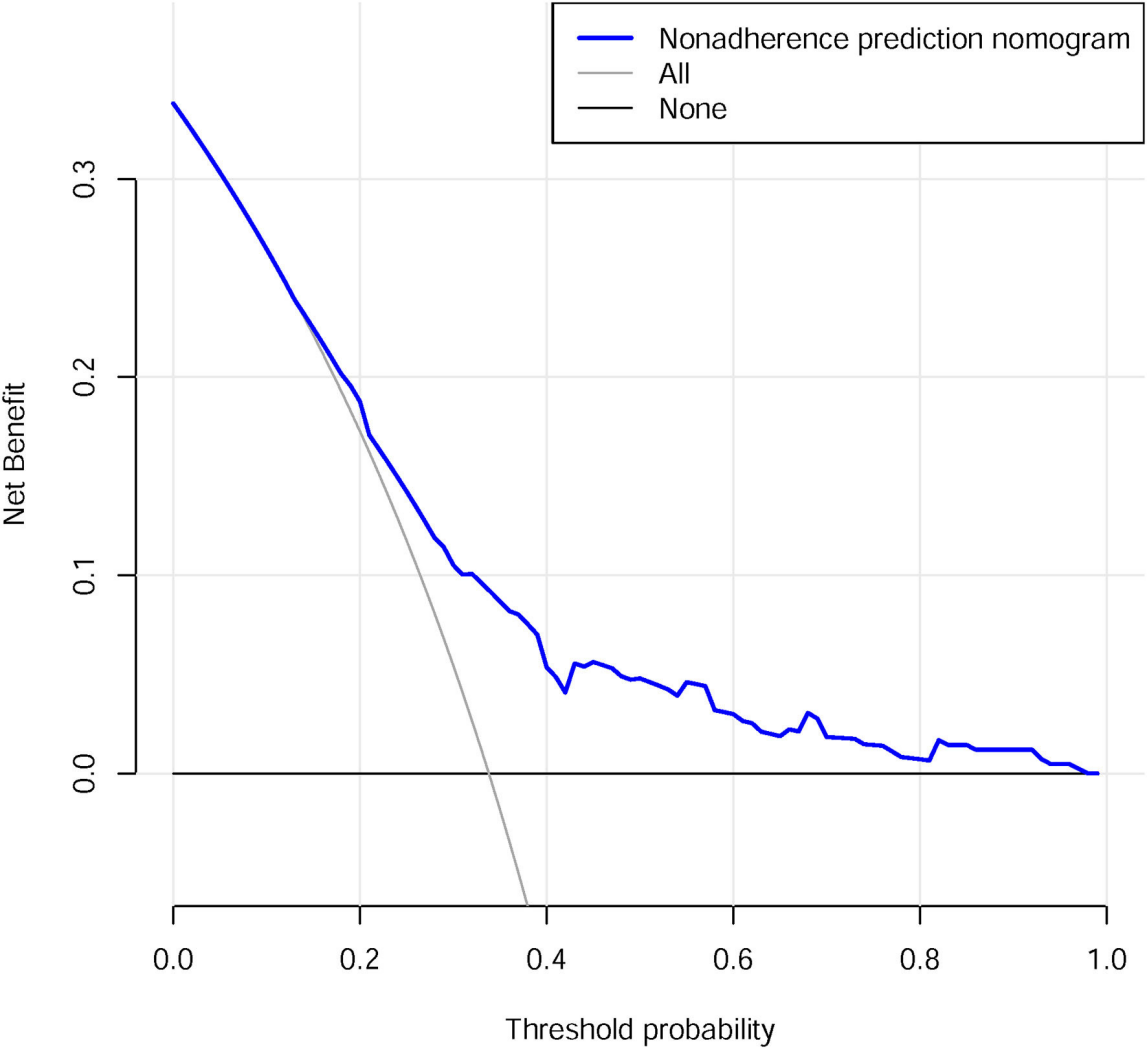
Decision curve analysis for the PROM nomogram.

The decision curve analysis for the PROM nomogram is presented in Figure 4. The decision curve showed that, if the threshold probability of a patient and a doctor was >13 and <34%, respectively, the use of this PROM nomogram to predict PROM risk added more benefit than the scheme. Within this range, net benefit was comparable with several overlaps, on the basis of the PROM risk nomogram.

## Discussion

Nowadays, nomograms are widely used as prognostic devices in oncology and medicine. Nomograms depend on user-friendly digital interfaces, increased accuracy, and more easily understood prognoses to improve patient management and clinical decision making. Our research was the first study to develop and validate a new nomogram to predict the risk of PROM in pregnant women with VVC. The covariates included age, regular perinatal visits, history of VVC before pregnancy, symptoms with VVC, cured of VVC during pregnancy, and bacterial vaginitis.

One large samples study in East China previously demonstrated that 15.3% of a total of 111,390 pregnant women suffered from PROM (17). In our study, about 33.8% of pregnant women with VVC suffered from PROM, which is double the findings of the previous study that the included population was not especially for VVC pregnant women. Therefore, it is suggested that PROM in pregnant women with VVC should be carefully managed. This nomogram suggested that avoiding pregnancy over the age of 35, regular perinatal visits, early detection, and treatment of VVC may be the key protective factors for PROM in pregnant women with VVC.

PROM and infection have reciprocal causation. Infection could generate PROM, and after PROM, the pathogen may cause a retrograde infection. When a pregnancy is infected, the inflammatory response may produce enzymes which could degrade the membrane, giving rise to rupture (18). Zhang et al. (19) notes that Keap-1/Nrf2 signaling pathway activation following oxidative stress is associated with patients with PPROM (preterm premature rupture of membrane). A related study (20) shows that high Caspase-3, AIF, and low Bcl-2 expression are risk factors for PROM. Infants in the PROM group experienced higher rates of infection, asphyxia, and jaundice.

Advanced maternal age, defined as age ≥35 years at estimated date of delivery, is considered to lead to a higher incidence of obstetric complications and adverse pregnancy outcomes (21). Consistent to this guide, our investigations indicated that advanced maternal age was a risk factor for PROM in gravidas with VVC. It is known that vaginal micro-ecology is closely related to immunological status, and that the body's defense for fighting virulent hyphae gradually decreases with aging. Many studies indicate that advanced maternal age is a strong independent risk factor for adverse outcomes (22–24). Nowadays, women are delaying childbearing to pursue educational and career goals in greater numbers than ever before; as a result, advanced maternal age is a common phenomenon. Despite a fall in the birth rate of the general population of the United States over the past three decades, the birth rate for women aged 33–55 years has risen (25). Clinicians should educate both patients and the public that there is a real danger for mother and child if individuals choose to delay reproduction (26).

To our surprise, regular perinatal visits, had the greatest effect on PROM in pregnant women with VVC. Regular and efficient antenatal visits lead to a high percentage of good outcomes for mothers and infants (27), such that most women visit the hospital for scheduled check-ups during gestation. However, owing to an uneven distribution of medical resources and the uneven development of urban and rural areas in China, some pregnant women with low levels of education or that live in remote mountain areas are not aware of the significance of antenatal care. The government should increase the spread of prenatal care to these individuals. Clinicians are more sensitive to the observation of symptoms; therefore, regular perinatal visits could lead to symptom identification and thus prevent the development of adverse events. Abnormal vaginal discharge has shown a significant association with the occurrence of PROM. Pregnant women who had an abnormal vaginal discharge are more likely to develop PROM (28, 29). Abnormal vaginal discharge is indicative of infection, and infection causes inflammation of the membrane leading to subsequent rupture (30, 31). The symptoms of VVC in pregnancy were similar to those in the non-pregnant state: pruritus vulvae, leucorrhoea with a peculiar smell, flush on the vulva, discomfort, thick cottage-cheese–like discharge associated with vaginal and vulvar pruritus, pain, burning, erythema; in addition, there were quite a few asymptomatic infected individuals. Our study implied that symptoms of VVC were more prone to lead to PROM. A recent study (8) also indicates that abnormal vaginal discharge (adjusted OR = 5.30, 95% CI = 2.07–13.52) is relevant to PROM. At least one vaginal discharge examination is recommended during pregnancy for those women who have no complaints, and this examination is inspected preferably in the first trimester (12). Once VVC is found, it should be treated without delay. The latest WHO guidelines on the management of vaginal discharge (32) recommend that a single dose (oral or vaginal) of azole is a simple and the best treatment for VVC. Here, we again emphasize the importance of antenatal care. Only regular visits could ensure identification and treatment in a timely manner.

Furthermore, history of VVC before pregnancy was also an essential factor for PROM. VVC is a public health problem, vaginal dysbacteriosis and hypoimmunity are the most common pathogenesis of VVC (33). Pregnant women have a 2-fold increase in the prevalence of vaginal colonization by candida species compared with non-pregnant women (2). One study reveals that the prevalence of VVC in pregnant women is 36.5% (34). During pregnancy, the balance of vaginal flora is easily disrupted and disequilibrium is triggered. Because of this susceptible constitution, attention should be paid to those pregnant women with a history of VVC before pregnancy. On the basis of this systematic review (5), recurrent VVC probably affects more than 130 million women in any given year, with a global annual prevalence of 3,871 per 100 000 females. This damaging effect of VVC should be made widely known. In clinical work, clinicians need to attach importance to the past medical history, and medical records should be regarded as essential information.

Bacterial vaginitis is common and caused by a disruption of the microbiological environment in the lower genital tract. In the US, the reported prevalence of bacterial vaginitis among pregnant women ranges from 5.8 to 19.3% (35). Bacterial vaginitis during pregnancy has been associated with adverse obstetrical outcomes including preterm delivery, early miscarriage, postpartum endometritis, and low birth weight (36). Here, our findings showed that bacterial vaginitis increased the risk of PROM for pregnant women with VVC; this may be owing to the concurrent effect, i.e., that bacterial vaginitis and VVC accelerate an inflammatory response. Thus, gravidas with VVC and bacterial vaginitis were susceptible to PROM.

Moreover, during the review of previous literature, some potential risk factors, such as, education level, BMI before pregnancy, malpresentation, and polyhydramnios showed a relationship with PROM. Education in this matter could protect against infection (37).One study associated with assisted reproductive technology observes that intracytoplasmic sperm injection and elevated BMI increase the risk of PROM (38); furthermore, polyhydramnios may result in PROM (39). In addition, Assefa et al. (40) found that gravida with a history of PROM were more prone to develop PROM. Nevertheless, when confounding factors were added, above all of these were not considered in the nomogram lastly.

This PROM risk prediction tool is concise and explicit, and assist clinicians with the early identification of patients at high risk of PROM. The covariates are objective indicators, but not subjective indicators. Moreover, the included information in this nomogram is for almost zero cost.

Lyu et al. (41) provided a model for predicting PPROM based on laboratory data, such as white blood cells, granulocytes, lymphocytes, and neutrophil lymphocyte ratio. Malchi et al. (42) implied that vaginal fluid urea and creatinine were indicators of PROM. Zhan et al. (43) found that routine blood tests were a good indicator for predicting PROM, and Liang et al. (44) suggested that a routine urine test had partial predictive value for PROM. These methods of predicting PROM require collection of blood or urine samples at the time of occurrence of PROM, not prior. In our study, the nomogram is calculated based on existing medical history, allowing prediction of PROM before it happens. Additionally, based on this prediction model, early interventions such as advice of regular perinatal examination and early detection and treatment of VVC will contribute to control the occurrence of PROM.

There were several limitations of our current study. First, the findings need to be externally evaluated in a greater number of pregnant women with VVC. In the future, larger prospective research involving multiple centers, as well as a larger number of patients, should be undertaken with longer periods of follow-up, to validate and improve the nomogram. Secondly, due to the limited number of samples, we did not study the PPROM group separately. PPROM is highly correlated with severe outcomes, and deserves to be investigated further. Certainly, PPROM population will be extracted in our following study.

This study developed a novel nomogram that was relatively accurate in assisting clinicians to assess the risk of PROM in pregnant women with VVC. Age, regular perinatal visits, history of VVC before pregnancy, symptoms with VVC, cured of VVC during pregnancy, and bacterial vaginitis were the factors closely related to PROM in this nomogram. Candida species, maternal and neonatal outcomes, and mechanisms may be the future research directions. In addition, with the development of technology, optical coherence tomography may be a potential tool in the prediction of PROM (45).

## Data Availability Statement

The raw data supporting the conclusions of this article will be made available by the authors, without undue reservation.

## Ethics Statement

The studies involving human participants were reviewed and approved by the Ethical Committee of the First Affiliated Hospital of Guangzhou University of Chinese Medicine. The patients/participants provided their written informed consent to participate in this study. Written informed consent was obtained from the individual(s) for the publication of any potentially identifiable images or data included in this article.

## Author Contributions

LY, JG, and SL: project development. LY, HW, and YL: data collection. HW, JG, and XL: data procession. LY and CZ: manuscript writing. SL: manuscript editing. All authors contributed to the article and approved the submitted version.

## Funding

This work was supported by the following funding agencies: the program of National Natural Science Foundation of China (Grant No. 81904239), the innovation and strength program of the First Affiliated Hospital of the University of Chinese Medicine (Grant No. 2019QN11), the program of Guangzhou Science and Technology Bureau (Grant No. 202102020541).

## Conflict of Interest

The authors declare that the research was conducted in the absence of any commercial or financial relationships that could be construed as a potential conflict of interest.

## Publisher's Note

All claims expressed in this article are solely those of the authors and do not necessarily represent those of their affiliated organizations, or those of the publisher, the editors and the reviewers. Any product that may be evaluated in this article, or claim that may be made by its manufacturer, is not guaranteed or endorsed by the publisher.
